# 3D‐Printed Soft Lithography for Complex Compartmentalized Microfluidic Neural Devices

**DOI:** 10.1002/advs.202101787

**Published:** 2021-06-24

**Authors:** Janko Kajtez, Sebastian Buchmann, Shashank Vasudevan, Marcella Birtele, Stefano Rocchetti, Christian Jonathan Pless, Arto Heiskanen, Roger A. Barker, Alberto Martínez‐Serrano, Malin Parmar, Johan Ulrik Lind, Jenny Emnéus

*Adv. Sci*. **2020**, *7*, 2001150

DOI: 10.1002/advs.202001150

In the originally published article, some information was missing in the acknowledgements. Please find the correct acknowledgements here:

J.K. and M.B. thank funding by BrainMatTrain European Union Horizon 2020 Programme (H2020‐MSCA‐ITN‐2015) under the Marie Skłodowska‐Curie Initial Training Network and Grant Agreement No. 676408. S.V. thanks funding by Training4CRM European Union Horizon 2020 Programme (H2020‐MSCA‐ITN‐2016) under the Marie Skłodowska‐Curie Innovative Training Network and Grant Agreement No. 722779. J.U.L. and C.J.P. would like to acknowledge The European Commission MSCA‐IF (798820), Lundbeck Foundation (R250‐2017‐1425) and The Independent Research Fund Denmark (8048‐00050) for their support. Lund University Bioimaging Centre (LBIC), Lund University, is gratefully acknowledged for providing experimental resources for SEM. Lotte Nielsen is acknowledged for the help with MALDI–TOF experiments, Boris Ignjatović for the help with video editing and 3D modeling, and Dr. Francesca Garbarino for the input on the initial drafts of the manuscript.

